# Platinum Complexes Can Bind to Telomeres by Coordination

**DOI:** 10.3390/ijms19071951

**Published:** 2018-07-03

**Authors:** Lina Saker, Samar Ali, Caroline Masserot, Guillaume Kellermann, Joel Poupon, Marie-Paule Teulade-Fichou, Evelyne Ségal-Bendirdjian, Sophie Bombard

**Affiliations:** 1 INSERM UMR-S 1007, Cellular Homeostasis and Cancer, 75006 Paris, France; linasaker@yahoo.com (L.S.); samarali83@yahoo.com (S.A.); carolinemasserot@hotmail.com (C.M.); guikell@gmail.com (G.K.); 2 Paris Descartes University, Paris Sorbonne Cité, 75006 Paris, France; 3 Paris Sud University, Paris-Saclay University, 91405 Orsay, France; Marie-Paule.Teulade-Fichou@curie.fr; 4 Laboratoire de Toxicologie-Biologique, Hôpital Lariboisière, 2 rue Ambroise Paré, 75475 Paris, France; joel.poupon@aphp.fr; 5 Institut Curie-Recherche, Bât. 112, Centre Universitaire, 91405 Orsay, France; 6 CNRS UMR918, Centre Universitaire, 91405 Orsay, France; 7 INSERM U1196, Centre Universitaire, 91405 Orsay, France

**Keywords:** telomeres, platinum complexes, cisplatin, G-quadruplex, TRF2

## Abstract

It is suggested that several compounds, including G-quadruplex ligands, can target telomeres, inducing their uncapping and, ultimately, cell death. However, it has never been demonstrated whether such ligands can bind directly and quantitatively to telomeres. Here, we employed the property of platinum and platinum-G-quadruplex complexes to target G-rich sequences to investigate and quantify their covalent binding to telomeres. Using inductively coupled plasma mass spectrometry, surprisingly, we found that, in cellulo, in the presence of cisplatin, a di-functional platinum complex, telomeric DNA was platinated 13-times less than genomic DNA in cellulo, as compared to in vitro data. On the contrary, the amount of mono-functional platinum complexes (Pt-ttpy and Pt-tpy) bound either to telomeric or to genomic DNA was similar and occurred in a G-quadruplex independent-manner. Importantly, the quantification revealed that the low level of cisplatin bound to telomeric DNA could not be the direct physical cause of TRF2 displacement from telomeres. Altogether, our data suggest that platinum complexes can affect telomeres both directly and indirectly.

## 1. Introduction

Telomeres are specialized nucleoprotein structures that protect chromosome ends from being recognized as double strand breaks. In humans, telomeres are formed by a complex six proteins, TRF1, TRF2, TIN2, POT1, RAP1, and TPP1, named the shelterin, which prevents activation of the DNA damage response (DDR), protects against Non-Homologous End Joining (NHEJ) or Homologous Recombination (HR) repair pathways, and assists telomere replication [1]. Among the shelterin components, two proteins that bind directly to the double strand telomeric DNA, telomeric repeat-binding factors 1 and 2 (TRF1 and TRF2), are important components for telomere maintenance [2,3]. TRF2 has a critical role in the inhibition of ATM (ataxia telangiectasia mutated) kinase DDR pathways, as well as in the NHEJ and HR mechanisms [4,5,6]. Thus, TRF2 gene inactivation is embryonically lethal because of ATM activation and telomere fusions. While TRF1 plays a distinct role on telomeres, it prevents replication fork stalling and ATR (Ataxia telangiectasia- and Rad3- related) activation. 

Since telomeric DNA consists of repeated G-rich sequences (TTAGGG)_n_, it can adopt G-quadruplex structures (G4), resulting from the stacking of consecutive guanine tetrads [7]. Many studies have provided evidence for the formation of such structures in vivo, including their visualization by G-quadruplex antibodies [8] and next-generation sequencing [9]. Computational analyses of the human genome [10,11] strengthens additional evidence. However, these studies have not only highlighted enriched G4 potential forming sequences in telomeres (5–20 kbp), but also in gene promoters and at the border between introns and exons [12]. These non-canonical secondary structures have been associated with a number of key biological processes, such as telomere maintenance, replication, transcription, splicing, and translation [13]. Since the G4 have been proposed to be a druggable target [14], many small synthetic molecules (G4-ligands) able to stabilize G4 have been designed and synthesized [7,15]. It has been reported that G4-ligands can affect telomere replication [16,17] and transcription and the stability of telomeric protein complexes. G4-ligands were first shown to inhibit telomerase activity. By blocking the 3’ single stranded extremity of telomeres structured in G4, G4-ligands prevent telomere extension by telomerase, leading to a progressive shortening of the telomeres and cell death [18]. Indeed, telomerase is a ribonucleoprotein, which is reactivated in 80% of cancerous cells and provide them with an unlimited proliferation due to its ability to maintain telomere length [19]. Besides this role, G4 ligands can also induce the displacement of TRF2 from telomeres to the nucleoplasm, without any apparent degradation of the TRF2 protein. This leads to telomere uncapping, cell cycle arrest, or apoptosis [20,21,22,23]. Metallic complexes are particularly suitable as G4-ligands [24,25] that can disturb telomere functions [26,27,28,29,30,31]. Among them, some Pt^II^ complexes bearing a labile halogen ligand (Cl^−^ or I^−^) are capable of interacting with the G4 by direct coordination to the nucleobase (adenines or guanines) and trap them irreversibly [29,32,33,34,35,36] after stacking to the structure [37]. *In cellulo*, we have shown that hybrid platinum complexes combining a platinum complex to a G4-ligand within the same molecule, MPQ (mono-para-quinacridine) [38], or PDC (pyridodicarboxamide) [35] induce a significant loss of TRF2 from the telomeres associated with telomere dysfunctions. The displacement of TRF2 caused by the hybrid platinum complexes was higher than the one seen with its individual components, demonstrating a synergistic effect between the coordinating Pt^II^ moiety and the G4-ligand to trigger telomere dysfunctions. All together, these results suggest that the binding of platinum complexes to telomeric DNA can prevent TRF2 binding to telomeres, and, thus, induce its delocalization from telomeres. The following data from the literature support this hypothesis. Unquestionably, in vitro studies have shown that cisplatin, a well-known chemotherapeutic agent that cross-links adjacent guanines [GG] in duplex DNA [39], binds efficiently to telomeric DNA with a two to three fold preference relative to genomic DNA, in accordance with the higher probability of finding adjacent guanines in telomeric DNA [40,41,42,43], and prevents TRF2 binding to telomeric DNA in vitro [44]. Moreover, a few studies have investigated the cellular effects of cisplatin [45] on telomeres, pointing out a possible interaction of this drug with telomeric DNA. We have shown that cisplatin induces TRF2 displacement from telomeres in HT1080 cells [38]. Telomere shortening has been observed after a short-term cisplatin treatment (HeLa and hepatoma cells) [46,47], suggesting an uncapping of telomeres. A gradual shortening of telomeres has also been observed in NER (nucleotide excision repair) deficient yeast cells [48], implying that the NER pathway involved in the reparation of cisplatin adducts [49] may play a critical role in the repair and maintenance of damaged telomeres [43]. This may be related to a recent study that has shown that NER is active at telomeres since it removes the photoproducts cyclobutane pyrimidine dimers faster than the bulk genome [50]. In contrast, other studies performed in three cell lines (neuroblastoma, HeLa, acute lymphoblastic T cell) showed no telomere length modifications independently from their initial length (4–80 kbp) [51].

**Figure 1:**
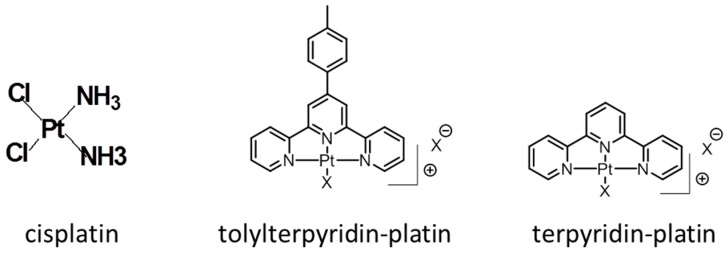
Chemical structures of cisplatin (cis-Pt), toly-terpyridin-platin, Pt-ttpy, and terpyridin-platin, Pt-tpy.

Herein, we quantified the amount of platinum adducts bound to telomeres following cell treatments with either cisplatin or two terpyridin platinum complexes, Pt-ttpy (tolyl terpyridin platinum complex), a G4-ligand shown to preferentially coordinate G4 structures [34], and its derivative, Pt-tpy (terpyridin platinum complex), a non-selective G4-ligand [34] (Figure 1). We showed that all platinum complexes employed bind to telomeric DNA, in cellulo. In detail, terpyridin platinum complexes showed an equal distribution on both telomeric and genomic DNA, while, conversely, cisplatin showed a slight preferential binding towards genomic DNA compared to telomeres. These results suggest a specific protection of telomeres from platination by cisplatin. We propose that this protection may result from a more efficient removal of cisplatin adducts by NER at telomere loci than at genomic loci. Importantly, we demonstrated that the amount of cisplatin bound to telomeres was not sufficient to explain TRF2 displacement by binding impairment. 

## 2. Results 

### 2.1. Validating Cellular Model and Telomere Purification Conditions

To study the association of platinum complexes to telomeres, we chose the ovarian cancerous cell line, A2780, and its cisplatin resistant counterpart, A2780cis. It has been previously reported that, in A2780cis cell lines, cisplatin resistance was mainly due to a reduced cellular cisplatin uptake and an increase of or more efficient repair mechanisms [52]. 

**Figure 2:**
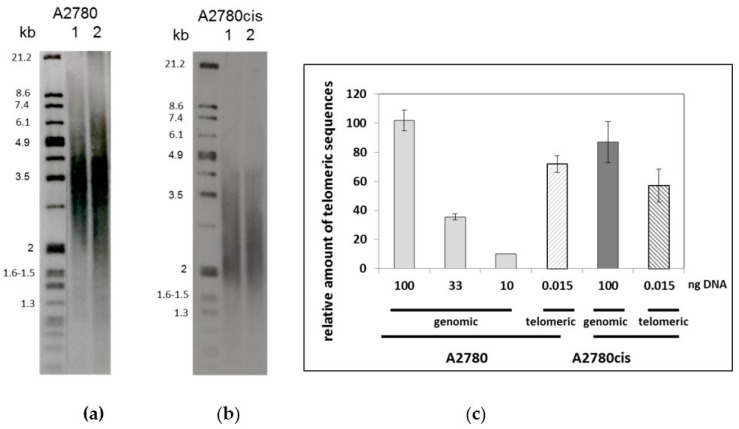
Telomere purification assay. Digested genomic DNA (4 µg) (lane 1) and isolated telomeres (2 ng) (lane 2) from A2780 cells (**a**) and A2780cis cells (**b**) were electrophoresed on a 0.6% agarose gel. Telomere restriction fragments were visualized using a luminescent telomeric probe. Size markers are shown on the left of each figure. (**c**) Relative enrichment in telomeres in the telomere purified fraction measured by qPCR compared to a range of non-digested genomic DNA.

Since telomeres represent less than 0.026% of the human genome, a large amount of genomic DNA was required to obtain enough telomeres to quantify a potential binding of platinum. Starting from 200 million cells (corresponding to 1.5 to 2 mg of genomic DNA), we obtained about 150 ng of telomeres following a purification method previously described and adapted [53]. To be noted, after digestion of genomic DNA by endonucleases, telomeres were purified by hybridization with a biotinylated telomeric C-rich strand oligonucleotide. The success of this procedure relies on the presence and full accessibility of the 3’G-rich overhang sequence located on the telomeres. Consequently, the integrity of the 3’G overhang after DNA extraction from treated and untreated cells was verified using the native hybridisation of the 3’G-overhang protocol by the C-rich telomeric radioactive probe [21]. Our results showed that our DNA extraction conditions preserved the 3’overhang intact and that the procedure did not modify the capacity of hybridization of the probe to the 3’G-rich overhang (Figure S1a). Moreover, we confirmed this result by UV melting temperature experiments. Since cisplatin GG adducts were known to affect the melting temperature of duplex oligonucleotides [54], we purified the cisplatin-GG adducts formed on the telomeric sequence (TTAGGG)_4_ by gel electrophoresis, as previously described [44], and determined the UV melting temperature of its duplex form compared to the one of the non-modified duplex (Figure S1b). As expected, the presence of a cisplatin DNA-adduct reduces the melting temperature from 56 to 42 °C and, importantly, these conditions remain fully compatible with the telomere purification procedure. The integrity of the purified telomeres was assayed by teloblot (Southern blotting and hybridization with a luminescent telomere specific probe) (Figure 2a,b). Of note, both cell lines displayed a slight difference in telomere length (3.5 kb for A2780 cells compared to 2.5 kb for A2780cis cells). 

The enrichment in telomeric sequences in the telomere fraction compared to genomic DNA was evaluated by two methods, namely teloblot and real-time telomere PCR [55,56]. By employing the first method, we found that the signal intensity corresponding to 2 ng of telomeres loaded on the gel was similar to that of 4 µg of genomic DNA. By employing the second method, the standard real-time telomere PCR assay [55,56], we verified that the SYBR green amplification signal was proportional to the genomic DNA amount (Figure 2c) and, consequently, to its telomere content. Moreover, the telomeric signal of A2780cis cells represents 85% of the telomeric signal of A2780 cells, which is consistent with the slightly shorter length of telomeres in the A2780cis cell line compared to the parental A2780 cells. Therefore, the relative amount of telomeric DNA in each purified telomere fraction could be evaluated: The signal intensity of 0.015 ng purified telomeres corresponds to that of 65–70 ng genomic DNA, indicating significant telomere purification (factor 5000) (Figure 1c).

### 2.2. Platinum Complexes Bind Covalently to Telomeres in Cellulo 

**Figure 3:**
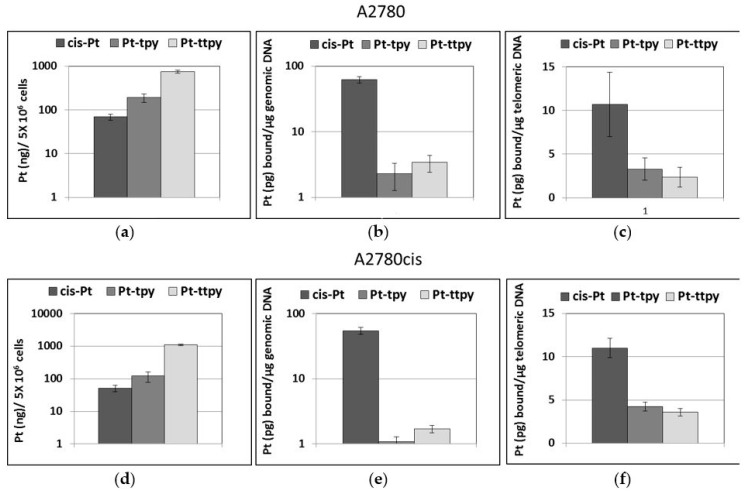
Quantification of the intracellular levels (left panel), of cisplatin, Pt-ttpy and Pt-tpy after 8 h treatment with 50 µM platinum complexes of A2780 cells (**a**–**c**) or A2780cis cells (**d**–**f**); of platinum bound to genomic DNA (middle panel) and bound to telomeric DNA (right panel).

The amount of cisplatin bound to genomic DNA after 96h cell treatments (0.6 µM and 6 µM for A2780 and A2780cis cells, respectively) was quantified in one of our previous study, giving 0.2 pg Pt/µg DNA for A2780 and 0.6 pg Pt/µg DNA for A2780cis [57]. This level of platinum could be easily detected because of the large amount of genomic DNA used for the quantification (20 µg). However, the telomeric DNA (150 ng) that can be purified was not sufficient to allow detectable levels of platinum bound. Indeed, after 96 h treatment by cisplatin in the above conditions, no platinum was detected in the purified telomeres from both cells lines, suggesting either an absence of platinum bound to them or rather the total amount of platinum was too low to be detected. To increase the amount of platinum complexes bound to DNA, we modified the conditions of the treatment by improving the time and the concentrations of platinum complexes. Cell treatments with 50 µM cisplatin for 8 h increased considerably the amount of cisplatin bound to genomic DNA: 62 pg Pt/µg DNA and 55 pg Pt/µg for A2780 and A2780cis cells, respectively (Figure 3b,e). The differences in cellular uptake and Pt binding to DNA in favor of A2780cis cells were consistent with the expected resistance phenotype [52,57,58,59], since it is known that cisplatin resistance in A2780cis cells is related to the lower uptake of the drug due to a mutation in the copper transporter, CTRl1. The fraction of living cells after 24 h of cisplatin treatment was 60 and 80% for A2780cis and A2780, respectively, whereas it was 100% after treatment by both terpyridin platinum complexes in both cell lines.

Since the 50 µM treatment allowed an increase by a factor 300 of the amount of cisplatin bound to genomic DNA, this condition was selected for platinum bound to telomere quantification for the three platinum complexes. While both terpyridin platinum complexes accumulated in treated cells at a higher level than cisplatin (in A2780, 3- and 10-fold accumulation and in A2780cis and 3- and 21-fold accumulation for Pt-ttpy and Pt-tpy, respectively) (Figure 3a,d), the amount of Pt-ttpy and Pt-tpy bound to genomic DNA was less important than that of cisplatin (in A2780, 18–27 fold less and A2780cis and 32–50 for Pt-ttpy and Pt-tpy, respectively) (Figure 3b,e). 

Of note, the higher accumulation of Pt-ttpy and Pt-tpy concomitantly with a lower DNA binding, as compared to cisplatin, seems to be a general trend since it has already been reported for many other platinum complexes [35,38,57,60,61]. These results suggest that either the adducts are more efficiently repaired than those generated by cisplatin and/or their covalent binding to DNA is less efficient [62]. Interestingly, in contrast to cisplatin, the amount of Pt-ttpy and Pt-tpy uptake was the same in both cell lines, but the amount of complexes bound to DNA was reduced in A2780cis cells, suggesting a more efficient repair mechanism in A2780cis cell lines as already reported [63]. 

Interestingly, we demonstrated for the first time that all three platinum complexes bind to telomeric DNA (Figure 3c,f). Independently from the cell line, a similar amount of Pt-ttpy and Pt-tpy was found at telomeric DNA and at genomic DNA; in contrast, the amount of cisplatin bound to telomeric DNA is 5-fold lower than to genomic DNA (Figure 3c,f). The preference of telomeric versus genomic DNA was thus dependent on the nature of the platinum complex. 

### 2.3. Platination of Telomeres by Cisplatin is not the Direct Cause of TRF2 Removal

**Figure 4:**
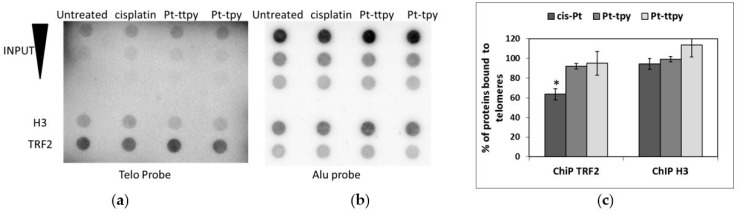
Quantification of TRF2 binding to telomeres by ChIP on A2780 cells treated with cisplatin, Pt-ttpy, or Pt-tpy for 8 h at 50 µM using anti-H3 and anti-TRF2 antibodies. The telomeric sequences immunoprecipitated by the anti-TRF2 or the anti-H3 antibodies were visualized after incubation of the dot blot membrane with a α ^32^P radiolabelled 800 pb telomeric probe (**a**); signal normalization was performed after hybridization of the same membrane with α ^32^P radiolabelled Alu sequences (**b**); two hundred nanograms of DNA were blotted for each ChIP sample. For the INPUT, 200, 100, and 50 µg of total DNA were blotted; (**c**) quantification of three ChIP experiments performed as in (**a**). Data were expressed as a percentage of the telomeric DNA signals in treated vs untreated cells. Quantitative values of the telomeric DNA signals are calculated as the ratio between the telomeric DNA signal precipitation and telomeric DNA signals in the INPUT for the same amount of blotted DNA. These values have been normalized to the amount of blotted DNA for each sample quantified by the non-specific Alu probe, following the formula: (telomere IP/telomere INPUT)/(Alu IP/Alu INPUT). (Means of at least three experiments) * Indicates a Mann and Withney test *p*-value *p* < 0.05 (GraphPad PRISM software, RITME, Paris, France).

Cisplatin has been shown to induce a 20-fold reduction of the capacity of TRF2 to bind telomeric DNA in vitro; [44] by interfering with the protein recognition sites and inducing a partial concentration and time dependent displacement of TRF2 from telomeres in cellulo [38]. Therefore, we wonder whether cisplatin adducts may also affect TRF2 binding to telomeres in living cells. Chromatin immunoprecipitation (ChIP) followed by dot-blot experiments were performed to quantify the amount of TRF2 associated with telomeres after cell treatments with 50 µM for 8 h. In these conditions, cisplatin treatment only induced a significant decrease in the binding of TRF2 to telomeres (30%) (Figure 4).

**Figure 5:**
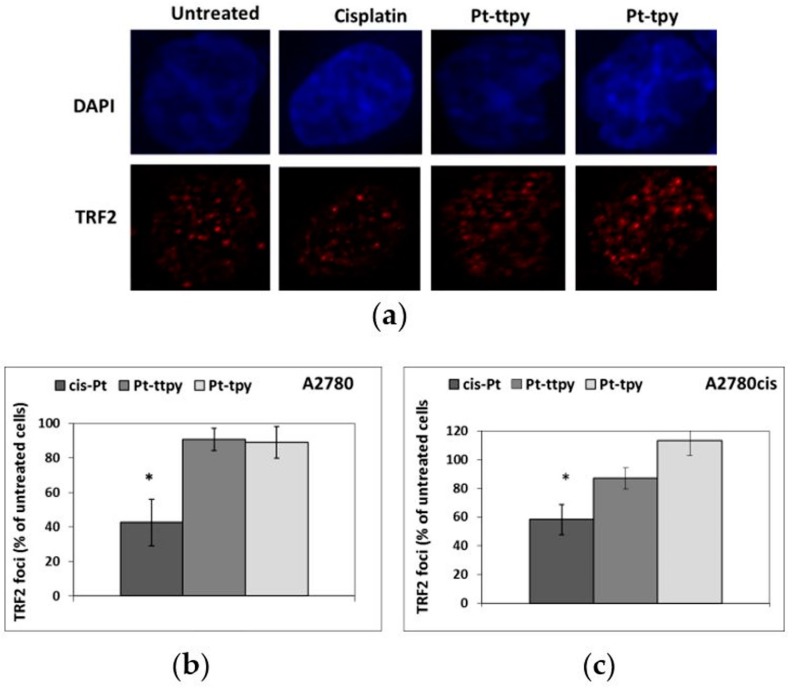
TRF2 foci quantification detected by immunofluorescence on A2780 and A2780cis cells treated with cisplatin, Pt-ttpy, and Pt-tpy for 8 h at 50 µM; (**a**) A2780 cells were processed for immunofluorescence using antibodies against TRF2; (**b**) % of TRF2 foci after A2780 cell treatments with cisplatin, Pt-ttpy, and Pt-tpy (**c**) % of TRF2 foci after A2780cis cell treatments with cisplatin, Pt-ttpy, and Pt-tpy (mean of at least three experiments). * Indicates a Mann and Withney test *p*-value *p* < 0.05 (GraphPad PRISM software, RITME, Paris, France).

These results were confirmed by immunofluorescence experiments showing a significant TRF2 foci decrease in cisplatin-treated cells only (Figure 5).

10 pg Pt was bound per µg of telomeric DNA (corresponding to 5 × 10^−14^ mole of Pt/3 × 10^−9^ mole of base), which gives a ratio of 0.00016 Pt bound per 100 bases. Considering that the amount of TRF2 bound to telomeres already estimated between 1.8 and 21 TRF2 molecules bound per 100 bases depending on the telomere length [64], our quantification implies that the amount of cisplatin bound to telomeres is too low to explain the removal of 30% of TRF2 from telomeres by a direct physical impairment of protein binding.

## 3. Discussion

That telomeres have been proposed as targets of G4-ligands was demonstrated primarily by indirect experiments, including the induction of telomere dysfunctions [20,21,22,23,26,27,28,29,30,35,38]. Direct evidence of telomere targeting has been obtained using a radiolabeled G4-ligand, ^3^H 360A [65]. Twenty five percent of ^3^H 360A was localized at the chromosomes ends. This G4 structure repartition has been confirmed by cellular immunofluorescence experiments using anti-G4 antibodies [8]. Platinum complexes, which were easily detected by ICP-mass, are alternative chemical tools of choice to study telomere targeting by drugs. Here, we showed that telomeres are directly targeted by platinum complexes. Surprisingly, the repartition of covalent platinum adducts between telomeric and genomic DNA did not depend on the capacity of platinum complexes to recognize G4, but rather depended on the platinum complex reactivity (mono- or di-functional). Pt-ttpy and Pt-tpy, as mono-functional platinum complexes, can coordinate only one guanine whereas cisplatin mainly crosslinks two adjacent guanines, GG [66]. A purely random genomic DNA would be expected to consist of 25% as guanine sites, a ratio that is the same than in the double stranded telomeric DNA repeat, TTAGGG/CCCATT. Therefore, since the level of Pt-ttpy and Pt-tpy bound to telomeric is similar to that bound to genomic DNA, it suggests that the binding of these drugs is dictated by the guanines’ densities. In contrast, the lower amount of cisplatin bound to telomeric DNA, as compared to genomic DNA, is unexpected given the previous in vitro results. Indeed, in vitro, platination of telomeric DNA by cisplatin is 2.6 times more efficient than genomic DNA data that have been correlated to the enrichment in GG sites in telomeric DNA as compared to genomic DNA (2.6-fold) [40,42,43,67]. Therefore, considering the in vitro data, the in cellulo telomeric platination is 13-fold less efficient than expected. 

The unexpected low ability of cisplatin to bind telomeric DNA compared to genomic DNA in vivo may be related to NER, the main pathway used to repair cisplatin adducts. Indeed, it was shown that NER participate actively in telomere integrity since the removal of photo-adducts at telomeres is dependent on the NER pathway, which operates at 1.5-fold faster than in the bulk genome [50]. Consequently, this suggests that terpyridin platinum complexes may be less efficiently repaired by NER at telomeres. One might also consider that the shelterin complex bound to telomeric sequences prevents the platination reaction, as compared to the histone environment in genomic DNA. However, this latter possibility is not supported by our data, with Pt-ttpy and Pt-tpy showing a similar platination of telomeric and genomic DNA. Since Pt-ttpy is a G4-ligand, it would have been expected that it would bind more efficiently to telomeres that are prone to form G4 [8] and to be bound by G4-ligands [65]. Our results show that Pt-ttpy covalently associates to telomeres as efficiently as Pt-tpy, indicating that this binding is independent of G4 recognition. Nevertheless, we cannot exclude the hypothesis that Pt-ttpy recognizes and stabilizes telomeric G4, but without trapping covalently this structure in a cellular context. This result highlights the fact that the covalent DNA trapping in vitro and in cellulo can also depend on the accessibility of the cross-linking sites. We have shown that the combination of a G4-ligand and a platinum complex within the same molecule improves TRF2 removal from telomeres, as compared to the individual components [35,38], and that cisplatin DNA-adducts prevent the binding of this protein in vitro [44]. Therefore, we analyzed if the amount of platinum complexes covalently bound to telomeric DNA was sufficient to provoke TRF2 removal from telomeres. However, only cisplatin, was able to induce a TRF2 delocalization in the conditions optimized for quantifying platinum complexes bound to telomeres. Interestingly, we showed that the amount of cisplatin bound to telomeric DNA is not sufficient to explain the displacement of TRF2 by a physical hindrance, suggesting that the delocalization of TRF2 observed is more likely due to alternative mechanisms as a biological regulation associated with the DNA damage response. These new observations, therefore, question the mechanism of telomeric protein delocalization by non-covalent G4-ligands [20,68,69]. Moreover, even if G4 ligands could potentially target other potential genomic G4 structures, the recent advances suggest that G4 ligands could be considered as promising therapeutic agents for tumors that are deficient in DNA damage repair. Therefore, the development of such ligands with improved selectivity for therapeutic purposes remains an actuality [12].

In conclusion, we demonstrated that, first, mono-functional platinum complexes are more prone to target telomeres than di-functional platinum complexes as compared to genomic DNA, and, second, that TRF2 delocalization from telomeres by DNA-targeting drugs could better be explained by an indirect mechanism involving a cellular signaling pathway triggered by the drug rather than a steric blockage due to the presence of many adducts generated at telomeres.

## 4. Materials and Methods 

### 4.1. Cell Lines and Culture Conditions 

A2780 and A2780cis cell lines (ATCC) were grown in RPMI medium supplemented with 10% fetal bovine serum and 3% of MIX (glutamine (2 mM), streptomycin (100 UI/mL), sodium bicarbonate (750 mg/mL)) and treated with 50 µM of platinum complexes, Pt-ttpy, Pt-tpy, or cisplatin, and incubated for 8 h or 0.6 and 6 µM cisplatin for 96 hin A2780 and A2780cis cells, respectively, at 37 °C under 5% CO_2_. 

### 4.2. Platinum Complexes 

Cisplatin was provided from Sigma. Pt-ttpy and Pt-tpy were synthesized following the procedure already described [34,70]. Aqueous solutions of cisplatin (1 mM) and Pt-tpy (5 mM) and DMSO solution of Pt-ttpy (6 mM) were prepared and conserved at −20 °C. Diluted solutions of each compound were freshly prepared. These conditions avoided an extensive exchange of the labile chloride against DMSO (less than 20%).

### 4.3. Platination of Double Stranded Telomeric DNA for Melting Temperature Analysis

(TTAGGG)_4_ was annealed with its complementary strand (CCCTAA)_4_ at 100 µM concentration in 0.1 M NaClO_4_., then incubated 18 h at 37 °C with 3 equivalents of cisplatin. An aliquot was used for 5’ ^32^P post-labelling using ^32^P γ-ATP. The platination products were isolated by denaturing gel electrophoresis. Radiolabelled platinated and non-platinated products were visualized after separation by gel electrophoresis using a Storm 960 Phosphorimager (Molecular Dynamics, Amersham Bioscience, Marolles-en-Hurepoix, France) together with Imagequant software for data processing. The non-radioactive products were revealed by UV shadowing. 

### 4.4. DNA Extraction

Genomic DNA was isolated from cells using the Kit DNAeasy^®^ blood and tissue (Qiagen, Courtaboeuf, France).

### 4.5. Platinum Quantification

The amount of platinum present in whole cell extracts from 5 × 10^6^ cells, DNA (20 µg) and purified telomere (150 ng) fractions were quantified by ICP-MS (Inductively Coupled Plasma Mass Spectrometry).

### 4.6. 3′ Overhang Assays

The non-denaturing hybridization assay to detect the telomere overhang was performed using the telomere repeat (CCCTAA)_3_CCC ^32^P-labelled probe. Briefly, 2.5 µg of extracted DNA was incubated overnight at 25 °C with the ^32^P-labelled probe, then electrophoresed in 0.7% agarose gel pre-stained with SYBR Green, as described [71]. The procedure allows detection of the amount of single-strand overhang available for hybridisation and the amount of loaded DNA. Both were quantified using the Storm 960 Phosphorimager (Molecular Dynamics, Amersham Bioscience, Marolles-en-Hurepoix, France) and the ImageQuant software program (Molecular Dynamics, Amersham Bioscience, Marolles-en-Hurepoix, France). The amount of radioactivity was normalised by the amount of corresponding DNA.

### 4.7. Purification of Human Telomeres 

Cells were grown in RPMI medium for 72 h (12 × 10^6^ cells for A2780 and 17 × 10^6^ cells for A2780cis cells) and treated by 50 µM of Pt-ttpy, Pt-tpy for 8 h. The protocol was adapted from [53]. Cells were recovered and DNA was extracted as above. The purified genomic DNA was subsequently digested overnight at 37 °C by HinfI and RsaI restriction enzymes. Each sample containing the digested DNA was incubated for hybridization with 150 pmole of biotinylated (CCCTTA)_4_ probe at 70 °C in 0.5× Saline Sodium Chloride-Sodium Citrate buffer (SSC) then allowed to cool to room temperature. Then, the streptavidin beads (Magnesphere, Promega, Charbonnières-les-Bains, France) in Denhardt’s solution were added to the samples and incubated on a rotating wheel at 4 °C with the annealed samples. The supernatant S1 containing genomic fragments and telomeric DNA not yet hybridized were recovered. Telomeric DNA was eluted twice with 150 μL water at 70 °C. The S1 fraction was re-hybridized with the probe attached to the beads, and the same steps as above were repeated. The DNA concentration was determined by measuring the absorbance at 260 nm using the Nano-Drop (1000 V) (Thermofisher, Villebon sur Yvette, France). 

### 4.8. PCR Telomere Enrichment Quantification

The relative telomere amount of the purified telomere fraction was determined by real-time PCR using the method described by Cawthon [55], and adapted for a LightCycler instrument [56]. It was not possible to normalize our telomeric signals with the reference. The genomic reporter gene, 36B4u, was not amplified in the purified telomeric fraction, suggesting an efficient purification even if this cannot exclude the remaining other genomic DNA regions.

### 4.9. Telomere Detection 

Aliquots of extracted DNA (4 μg) were digested overnight at 37 °C with RsaI and HinfI restriction enzymes (New England Biolabs, Evry, France). Digested DNA and purified telomeres were separated by agarose gel electrophoresis, and then transferred under denaturing condition to a nylon membrane by Southern blotting. Telomere length was then estimated using the “Telo TAGGG Telomere Length Assay” kit (Roche, Basel, Switzerland).

### 4.10. Immunofluorescence

After a 8 h treatment with platinum complexes, cells were fixed with 4% paraformaldehyde, permeabilized in a buffer containing 20 mM Tris-HCl pH = 8, 50 mM NaCl, 3 mM MgCl_2_, 300 mM Sucrose, 0.5% TritonX-100, and incubated in a blocking solution containing 5% BSA/PBS for 1h at room temperature before incubation with a mouse telomeric protein TRF2 antibody (4A794, Upstate, NY, USA) for 2 h at room temperature. The samples were then washed in PBS and incubated with a goat anti-mouse IgG secondary antibody TRITC (tetramethylrhodamine)-conjugated (Thermofischer)). Nuclei were counterstained with 4’,6-diamidino-2-phenylindole, Vectashield (DAPI). Three-dimension images (composed of 40 to 80 planes of 0.3 µm) were acquired using an inverted microscope with Epi-fluorescence attachment (Nikon Eclipse TE-2000 E) (Nikon, Champigny sur Marne, France). The number of TRF2 foci in each nucleus was counted using the Image J software program after a two-dimensional projection of three-dimension images. 

### 4.11. ChIP (Chromatin Immunoprecipitation)

Cells were collected after fixation with formaldehyde, and lysed already as described [38]. The DNA of the nucleus was sonicated to obtain fragments of 1 kbp. Thirty µL were conserved in order to quantify the number of telomeric sequences before immuno-precipitation (INPUT). Immunoprecipitation was then performed with an anti-TRF2 polyclonal antibody (IMG-148A, IMGENEX). Two hundred ng of the immunoprecipitated DNA and INPUT were blotted onto a Hybond-XL membrane (Ge HealthCare, Amersham Bioscience, Marolles-en-Hurepoix, France). The telomere sequences were detected using an 800 bp telomere repeat (TTAGGG) ^32^P labelled probe obtained after digestion of the pUC Telo2 plasmid [72] by EcoRI and BamHI and radiolabelling by random priming using dCTP [α^32^P], TAGGGTTA/TAACCCTA (Eurogentec, Liege, Belgium) as primers and Klenow polymerase (Fermentas, Thermofisher, Villebon sur Yvette, France)). The Alu sequences were detected using a ^32^P labelled Alu probe that was obtained after the digestion of the pTopo Alu-AII plasmid (obtained after amplification of human genomic DNA with tgaaaccccgtctctactaaaaa and gtctcgctctgtcgccca primers, then cloned in pGEM-T vector (Promega, Charbonnières-les-Bains, France)) by EcoRI and radiolabelled by random priming using dCTP [α^32^P], the hexanucleotide mix (Roche, Boulogne Billancourt, France) as primers, and Klenow polymerase (Fermentas). The membranes were first hybridized with the telomere probe, and the amount of radioactivity was quantified using the Phosphorimager and ImageQuant software. The membranes were then dehybridized in boiling water containing 1% SDS, and were then hybridized with the Alu probe; the amount of radioactivity was quantified using the Phosphorimager and ImageQuant software. Fold enrichment of the immunoprecipitated fraction was calculated as the ratio between telomeric DNA signals after precipitation and telomeric DNA signals in the total INPUT DNA for the same amount of blotted DNA (200 ng). The values were normalized to the Alu signal in the immunoprecipitated and INPUT fractions for each condition using the (telomere IP/telomere INPUT)/(Alu IP/Alu INPUT) formula. The % of TRF2 bound to telomeres was given as a function of TRF2 bound in treated cells/TRF2 bound in untreated cells.

## Supplementary Materials

Supplementary materials can be found at http://www.mdpi.com/1422-0067/19/7/1951/s1.

## Abbreviations

Pt-ttpy	Tolylterpyridin-platin
Pt-tpy	Terpyridin-platin
TRF2	Telomeric repeat-binding factor 2
ChiP	Chromatin-immunoprecipiration
