# Supplementary material for: Platinum Complexes Can Bind to Telomeres by Coordination
Source: Int J Mol Sci. 2018 Jul 3;19(7):1951. doi: 10.3390/ijms19071951 (PMC6073198; doi:10.3390/ijms19071951)
Supplement: Supplementary file 1 [file ijms-19-01951-s001.pdf]

Supplementay Material

Platinum complexes can bind to telomeres by coordination

Lina Saker[1,2], Samar Ali[1,3], Caroline Masserot[1,3], Guillaume Kellermann[1,2], Joel Poupon[4], Marie-Paule Teulade-Fichou[3],[5,6,7], Evelyne Ségal-Bendirdjian[1,2,3]* and Sophie Bombard[1,2,3,5,6,7]*

[1] INSERM UMR-S 1007, Cellular Homeostasis and Cancer, Paris, France.
[2] Paris Descartes University, Paris Sorbonne Cité, Paris, France.
[3] Paris Sud University, Paris-Saclay University, Orsay, France.
[4] Laboratoire de Toxicologie-Biologique, Hôpital Lariboisière, 2 rue Ambroise Paré, 75475 Paris. France
[5]Institut Curie-Recherche, Bât. 112, centre Universitaire, 91405 Orsay, France
[6]CNRS UMR918, Centre Universitaire, 91405 Orsay, France
[7]INSERM U1196, Centre Universitaire, 91405 Orsay, France
* Correspondence: sophie.bombard@curie.fr; Tel.: +33 1 6986 3189 and evelyne.segal-bendirdjian@inserm.fr

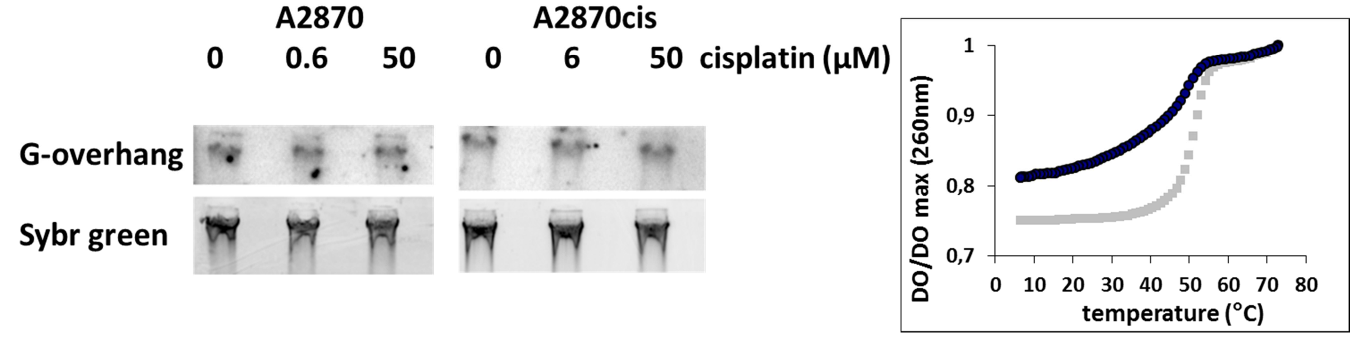

a b

Figure S1: Integrity of the 3'G-overhang and effect of a cisplatin GG adduct on its hybridization efficiency with the C-rich telomeric sequence. a) Telomeric single-stranded overhang signal hybridized with a the 32P radiolabelled (CCCTTA)$_4$ and the corresponding Sybr green signal for DNA quantification of A2780 and A2780cis cells treated with cisplatin at different concentrations for 96 or 8h. b) Melting temperature of (TTAGGG)$_4$/(CCCTTA)$_4$ duplex bearing (blue circle) or not (grey square) a cisplatin GG adduct in 50mM NaCl.
